# Mice lacking global *Stap1* expression do not manifest hypercholesterolemia

**DOI:** 10.1186/s12881-020-01176-x

**Published:** 2020-11-23

**Authors:** Babunageswararao Kanuri, Vincent Fong, April Haller, David Y. Hui, Shailendra B. Patel

**Affiliations:** 1 Division of Endocrinology, Diabetes and Metabolism, University of Cincinnati, Cincinnati, OH USA; 2 Department of Pathology, University of Cincinnati, Cincinnati, OH USA

**Keywords:** STAP1, Autosomal dominant familial hypercholesterolemia, Familial hypercholesterolemia 4, B-cells, Western diet, Fast performance liquid chromatography

## Abstract

**Background:**

Autosomal dominant familial hypercholesterolemia (ADH; MIM#143890) is one of the most common monogenic disorders characterized by elevated circulatory LDL cholesterol. Initial studies in humans with ADH identified a potential relationship with variants of the gene encoding signal transducing adaptor family member protein 1 (STAP1; MIM#604298). However, subsequent studies have been contradictory. In this study, mice lacking global *Stap1* expression (*Stap1*^*−/−*^) were characterized under standard chow and a 42% kcal western diet (WD).

**Methods:**

Mice were studied for changes in different metabolic parameters before and after a 16-week WD regime. Growth curves, body fats, circulatory lipids, parameters of glucose homeostasis, and liver architecture were studied for comparisons.

**Results:**

Surprisingly, *Stap1*^*−/−*^ mice fed the 16-week WD demonstrated no marked differences in any of the metabolic parameters compared to *Stap1*^*+/+*^ mice. Furthermore, hepatic architecture and cholesterol content in FPLC-isolated lipoprotein fractions also remained comparable to wild-type mice.

**Conclusion:**

These results strongly suggest that STAP1 does not alter lipid levels, that a western diet did not exacerbate a lipid disorder in *Stap1* deficient mice and support the contention that it is not causative for hyperlipidemia in ADH patients. These results support other published studies also questioning the role of this locus in human hypercholesterolemia.

**Supplementary Information:**

The online version contains supplementary material available at 10.1186/s12881-020-01176-x.

## Background

Familial hypercholesterolemia (FH) is a widely prevalent congenital metabolic disorder characterized by substantially elevated circulatory low density lipoprotein cholesterol (LDL-C) and accelerated cardiovascular events [1, 2]. Autosomal dominant inherited hypercholesterolemia (ADH) accounts for a major proportion of the FH cases worldwide with mutations most commonly reported in low density lipoprotein receptor *(LDLR)*, apolipoprotein B *(APOB)*, and proprotein convertase subtilisin/kexin type 9 *(PCSK9)* [3–5]. However, a number of patients classified as having familial hypercholesterolemia 4 (FH4), demonstrate the ADH phenotype despite having no mutations in these genes [6]. Initial studies involving these patients reported mutations in genes coding for signal-transducing adaptor family member 1 (STAP1) [6–8].

STAP1 (MIM#604298), also called B-cell antigen receptor downstream signaling 1 protein (BRDG1) or stem cell adaptor protein 1, was first discovered in immune cells with the highest expression documented in appendix, lymph nodes, and spleen [7]. The gene encoding STAP1 protein is located on chromosomal region 4q13.2 with 10 exons in the protein coding region [7]. Its relationship to lipid homeostasis was first suggested in 2007 in a clinical study finding a correlation between increased levels of circulatory triglycerides and *STAP1* gene expression in leukocytes [9]. Later, in 2014, Fouchier and colleagues discovered that variants of the *STAP1* gene were associated with the FH phenotype in a linkage analysis of a Dutch family with FH4 and suggested robust support for genetic causality [6]. Subsequent studies reported *STAP1* gene variants observed in such FH patients also manifested significant cardiovascular events [10–12]. However, other studies found no association between *STAP1* variants and hypercholesterolemia [13–16]. An analysis of seven families with FH phenotype failed to observe the co-segregation of four rare predicted pathogenic variants of *STAP1* [17]. Loaiza et al more recently demonstrated no marked changes plasma lipid profiles of carriers of *STAP1* variants compare to controls as well as in a mouse model [18]. Therefore, we sought out to understand the STAP1 role by using a mouse knockout model, to ask if loss of STAP1 would lead to hyperlipidemia.

In this study, mice lacking global expression of *Stap1* gene *(Stap1*^*−/−*^*)* were characterized for various metabolic parameters on standard diet and after 16 weeks of western diet (WD) regime. The present work presents an independent characterization of *Stap1*^*−/−*^ mice to that studied by Loaiza et al with some differences between models and confirms the argument against STAP1 having a role in causing FH.

## Methods

### Knockout mice and diets

Heterozygous *Stap1* KO mice (*Stap1*^*+/−*^) on C57BL/6 N background generated through reporter-tagged insertion were imported from Wellcome Trust Sanger Center, via EUCOMM [19–22]. The ‘knockout first’ tm1a (*Stap1*^*tm1a(KOMP)Wtsi*^) mutation bears the IRES:lacZ trapping cassette and a floxed promoter-driven neo cassette inserted into the intron of the gene in order to disrupt the gene function [22]. Brother-sister matings of the heterozygotes generated homozygous knockout mice (*Stap1*^*−/−*^) and corresponding WT (*Stap1*^*+/+*^) littermate controls. Tail snip genotyping was performed to assess the genotype of each mouse before recruiting into experiments; amplimers of 266 bp and 236 bp correspond to *Stap1*^*+/+*^ and *Stap1*^*−/−*^, respectively (Table). Mice were fed with irradiated standard rodent-chow diet (CD; Envigo 7912) [23] and water ad libitum, with equal light and dark cycles. Baseline measurements were performed in 10 to 11 week-old male and female mice on rodent chow and then placed on a high-cholesterol western diet (WD; Envigo TD.88137) for another 16 weeks [24]. Chow and western diets provided 17 and 42% kcal energy from fats [23, 24] and were procured from Harlan Teklad, Madison, WI, USA. All animal studies were approved by the Institutional Animal Care and Use Committee at the University of Cincinnati (Protocol No. 16–04–19-01). Terminal blood samples were obtained under full inhaled isoflurane anesthesia. For all studies, except where indicated, each group consisted of 6 mice, based upon prior data that show the power to detect 20% differences for the most variable procedure (OGTT) where the Power needed is *n* = 5, but we included and extra mouse to ensure Power was not due to loss of a mouse for any inadvertent loss. The investigators were not blinded to the samples or mouse genotypes, as there are no datasets collected which required subjective analyses. However, all mice were group housed and each cage contained mice of both genotypes to minimize bias and variation. Mice were sacrified by CO2 inhalation, followed by cervical dislocation and bilateral thoracotomy.

### Measurement of whole-body composition

Whole-body fat and lean mass were measured in live mice using ECHOMRI™-100H whole body composition analyzer (Echo Medical Systems, Texas, USA) according to the manufacturer’s specifications. Absolute weights (g) of fat and lean mass of each mouse were used to calculate their % changes [(absolute fat or lean mass weight (g)/whole body weight(g))*100], a widely employed method to determine the diet induced obesity phenotype.

### Oral glucose tolerance test (OGTT)

For the OGTT experiment, the experimental mice were initially acclimatized in the procedure room overnight followed by 4 h fast period in the next day morning. After baseline blood glucose measurements, 50% Dextrose (Cat# D16–1; ThermoFisher) in 0.9% saline was administered via oral gavage at a dose of 2 g/kg of body weight [24]. Time dependent changes in blood glucose were measured via tail vein bleeds at 0, 15, 30 and 60 min using Accu-Chek Nano electronic glucometer (Roche Applied Science, Indianapolis, IN, USA).

### Fast performance liquid chromatography (FPLC)

Lipoprotein separation for total cholesterol estimation in plasma samples was performed using FLPC as described previously [24]. Briefly, blood was collected from 4 h fasted mice through the submandibular plexus and then centrifuged at 2500×g for 10 min at 4 °C. The isolated plasma was then measured for basal total cholesterol to exclude any outliers (to ensure pooling was performed between comparable samples) and 200 μL of pooled plasma from each group was loaded onto the Akta pure FPLC to separate different lipoproteins by column chromatography (10/300GL, superpose 6). A total of 52 fractions with volume of 500 μL each were collected and analyzed for cholesterol content using an enzymatic kit as detailed below.

### Plasma biochemistry

Plasma triglycerides and cholesterol were estimated using Infinity Triglyceride (TR22421) and Cholesterol (TR13421) calorimetric kits (Thermo Fisher Scientific, Middletown VA, USA) per manufacturer instructions. For estimation of tissue lipids after the western diet phase, livers from *Stap1*^*+/+*^ and *Stap1*^*−/−*^ mice were homogenized, normalized for equal protein content, and then measured for total cholesterol and triglycerides using the same calorimetric kits used for plasma samples.

### Tissue harvesting and histology of liver

Mice were euthanized for terminal tissue collection at the end of 16 weeks WD regime. Four-hour pre-fasted mice were euthanized by CO_2_ exposure and bilateral thoracotomy. Liver, spleen, and kidneys were blotted dry, weighed, and then flash frozen in liquid N_2_. A small portion of liver was rinsed in chilled PBS (pH -7.4) and fixed in 10% neutral buffer formalin (5725; Fisher Scientific, Pittsburg, PA, USA) for paraffin embedding and hematoxylin and eosin (H&E) staining [25]. About 5 μm of paraffin embedded sections were cut, processed in graded volumes of ethanol, and then stained with H&E. Images of the stained liver sections were captured at 20x and 40x objective lens magnification using Olympus BX61 microscope.

### QPCR analysis of gene expression

To determine the tissue expression profile of *Stap1*, total RNA were isolated from different tissues of *Stap1*^*+/+*^ mice using Qiagen RNeasy Mini Kit (74,104; Qiagen Inc., Germantown, MD, USA). Isolated RNAs were reverse transcribed to cDNA using SuperScript First-Strand Synthesis kit (11904–018; Invitrogen, Carlsbad, CA, USA) and then assessed for the *Stap1* gene expression on Applied Biosystems 7300 Real-Time PCR system (Applied Biosystems, Foster City, CA, USA). Each sample was run in triplicates using power SYBR Green PCR master mix (4,367,659; Applied Biosystems, Foster City, CA, USA). Comparative C_T_ method was used for quantitation post data normalization to *Atp5po* expression as the house keeping gene to compensate the variations between input RNA amounts [25]. The list of different primers used for PCR experiments was tabulated in the Table S1 (Additional file 1).

### Statistical analyses

An unpaired t-test (without or without Welch’s corrections) or Mann-Whitney U test was performed to assess significant between *Stap1*^*−/−*^ and *Stap1*^*+/+*^ mice within each sex.

## Results

### Validation of *Stap1* gene deletion

**Fig. 1:**
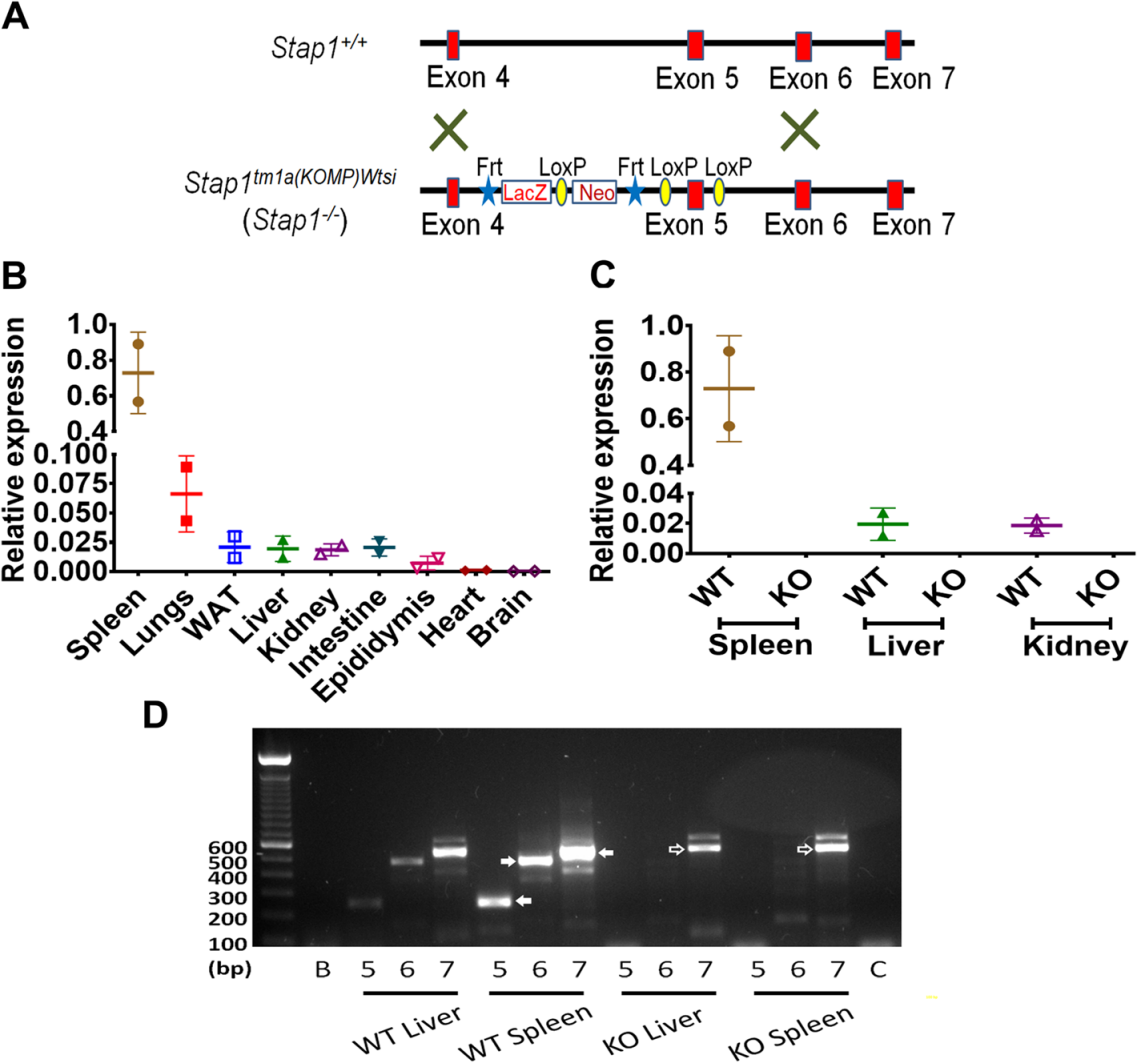
Verification of global deletion of *Stap1* gene expression. Panel **a** shows the gene targeting details for the generation of *Stap1*^*−/−*^ mice. This construct is a ‘knockout-first’, which can be converted to a conditional, upon deletion of the LacZ-Neo cassette. The mice used in this study remain as knockout and retain this cassette. A tissue survey of Stap1 mRNA was performed using qPCR in tissues collected from *Stap1*^*+/+*^ mice (panel **b**, *n* = 2). Note that compared to a house-keeping gene expression (see Methods), only spleen and lung showed some expression with very low levels detected in other tissues. *Stap1*^*−/−*^ mice (KO) showed no detectable qRT-PCR products (panel **c**, n = 2–3) compared to wild-type mice (WT) in spleen, liver or kidney. To further confirm a lack of legitimate transcript expression, RT-PCR analysis of exon splicing for exons 2–5, 2–6, and 2–7 in liver and spleen was performed (panel **d**). The labels ‘5, 6, and 7’ for each tissue indicates the location of the reverse primers targeting exons 5, 6, or 7 respectively, used with a forward primer located in exon 2. Spleen mRNA from WT tissues amplified the correct expected size of product and size increments (exon 2–5; 251 bp, exon 2–6; 482 bp, and exon 2–7; 536 bp indicated by filled arrowheads). No products were noted when exons 2–5, or 2–6 were used for KO spleen mRNA. A slightly larger product was noted for exons 2–7 (indicated by open arrowhead), though on sequencing this was found to be a non-specific product from mis-priming (see Text). Error bars denote ±1 SD

The targeting strategy for generation of global *Stap1* knockout mice is depicted in Fig. 1a. Measurement of transcript levels of *Stap1* in WT mice by qPCR revealed gene expression in a variety of tissues with the highest levels demonstrated in spleen (Fig. 1b). The complete absence of *Stap1* expression was confirmed with qPCR in *Stap1*^*−/−*^ mice liver, kidney, and spleen (Fig. 1c). RT-PCR analysis of exons 2–5, 2–6, and 2–7 using total RNA isolated from livers and spleens demonstrated sequential exon splicing of exons 5, 6, and 7 in *Stap1*^*+/+*^ mice and no products in *Stap1*^*−/−*^ mice (Fig. 1d). The single slightly larger amplicon for exon 2–7 in *Stap1*^*−/−*^ spleen (Fig. 1d, open arrowhead) was sequenced and demonstrated an off-target product on chromosome 2 (Additional file 2).

### Baseline characteristics

**Fig. 2:**
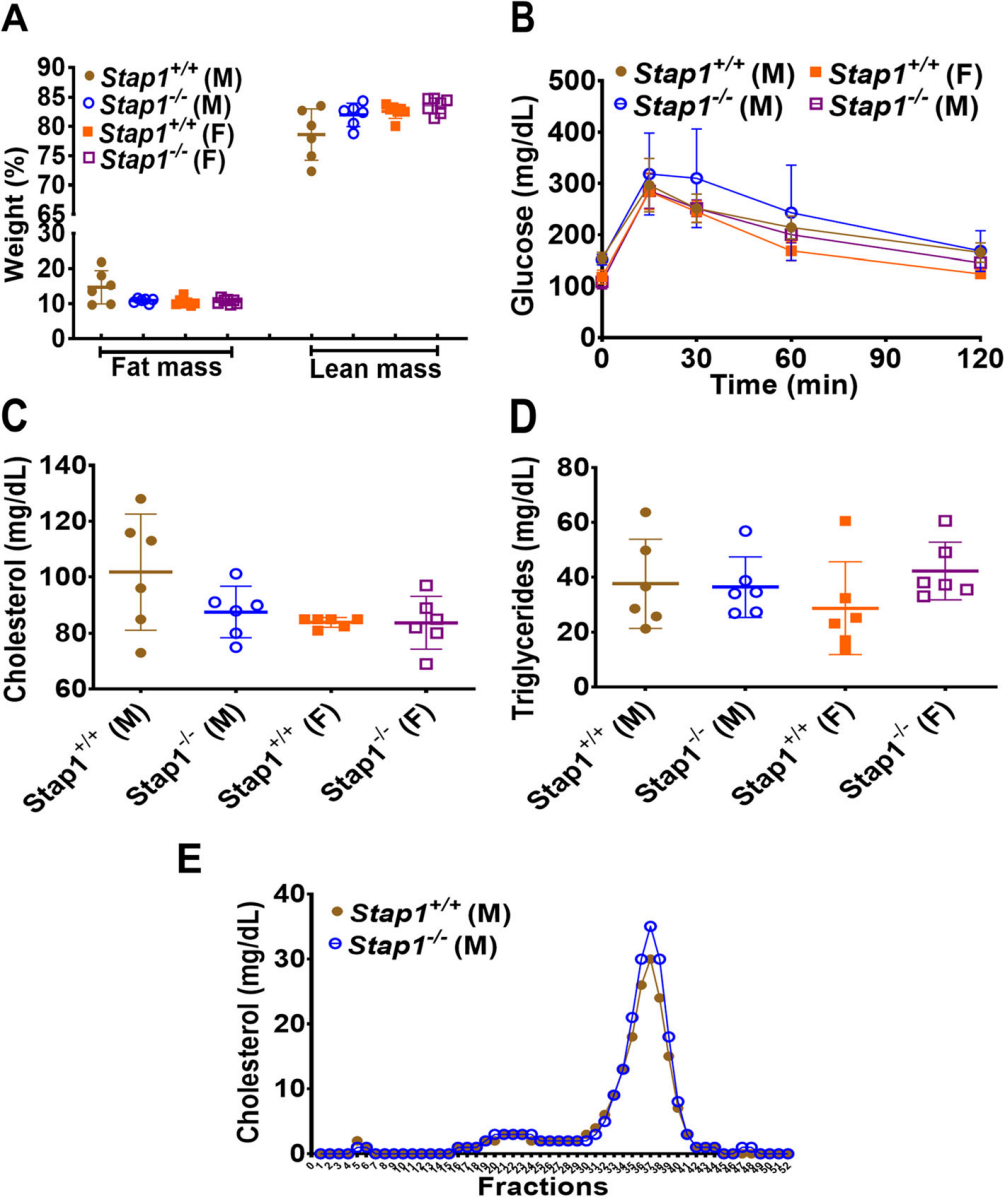
Baseline metabolic parameters in chow-fed *Stap1*^*−/−*^ and control mice. Panel **a** shows the starting body composition of mice at age 5–6 weeks on a chow diet (*n* = 6–7). Starting lean or fat masses between wildtype and knockout mice were comparable. Glucose tolerance (Panel **b**, n = 6), plasma cholesterol (Panel **c**, n = 6), triglycerides (Panel **d**, n = 6) were also comparable and showed no statistical differences. Panel **e** shows the FPLC cholesterol profiles in 10–11 weeks old male *Stap1*^*+/+*^ and *Stap1*^*−/−*^ mice and was also indistinguishable. Note that the knockout mice do not show any differences in the VLDL (fractions 3–6), or LDL (fractions 20–28) ranges. Error bars denote ±1 SD

**Fig. 3:**
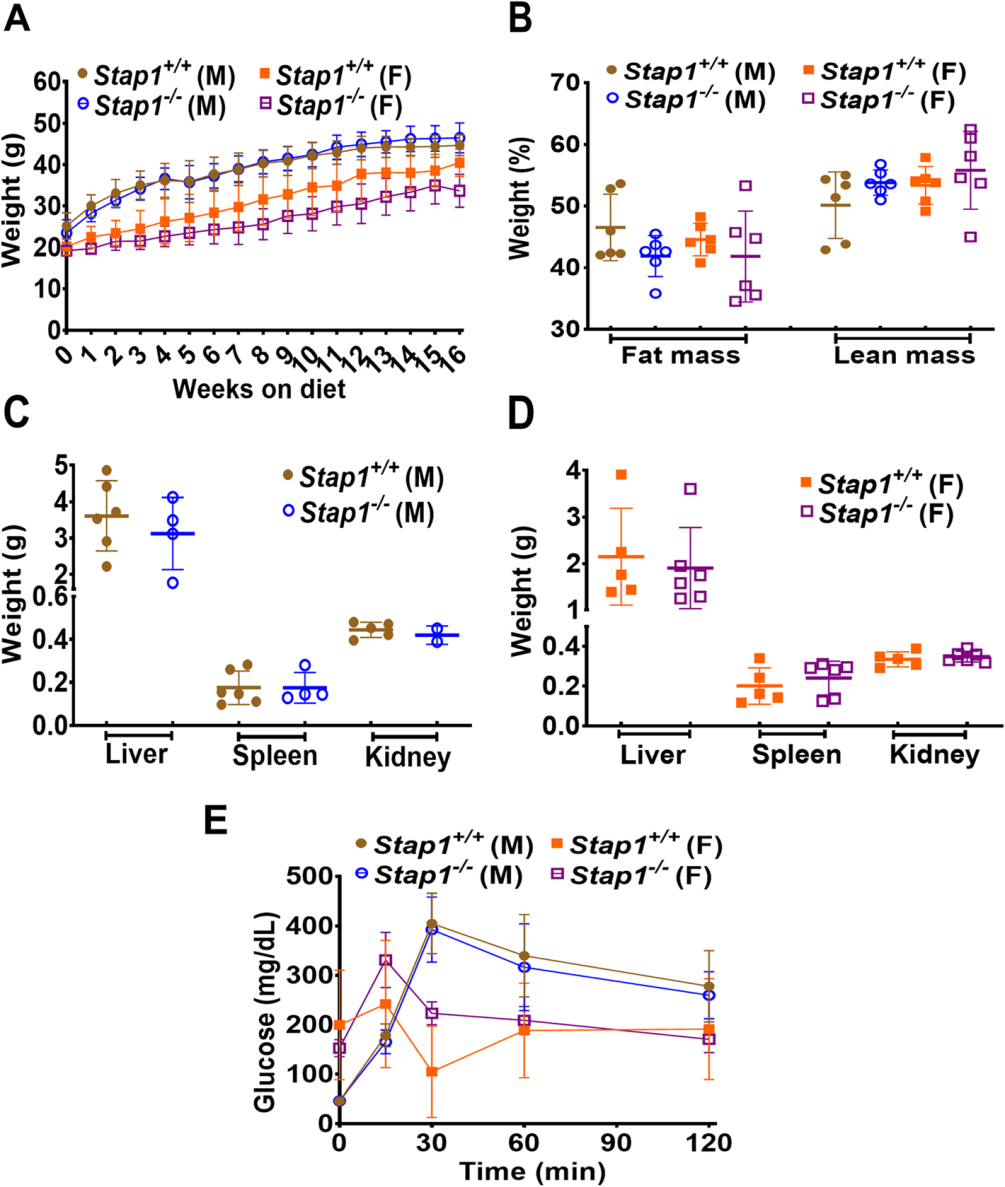
Effect of western diet on growth parameters and body composition in mice lacking *Stap1* gene. Panel **a** shows the growth curves in wild-type and knockout mice and no statistical differences were discernable in the patterns of weight gain on the high caloric diet (n = 6). Neither the percent fat or lean mass were altered in *Stap1*^*−/−*^ mice compared to age and sex-matched wildtype mice on a western diet (panel **b**, n = 6). No changes in weights of liver, spleen or kidneys were seen in male (panel **c**, *n* = 4–6) or female mice (panel **d**, *n* = 5–6) were noted. No changes in glucose tolerance tests were noted between knockout and wildtype controls either (panel **e**, n = 6). In case of male *Stap1*^*−/−*^ mice, kidney weights of only 2 mice were considered for analysis

Baseline measurements on standard chow diet were assessed at 10–11 weeks of age and body weights (Fig. 3a), whole-body fat (Fig. 2a) and lean mass (Fig. 2a) were similar between *Stap1*^*+/+*^ *and Stap1*^*−/−*^ mice. Further biochemical assessment found no significant differences in glucose tolerance (Fig. 2b), plasma total cholesterol (Fig. 2c), or plasma triglycerides (Fig. 2d) between knockout mice and wildtype mice. Lipoprotein cholesterol profiles in male knockout and wild-type mice were analyzed by FPLC (Fig. 2e) and were also found to be unaffected by the loss of *Stap1*.

### Effects of western diet (WD)

To determine the role of *Stap1* under metabolic challenge, mice were fed western diet (42% kcal from fat). Prior to the commencement of the western diets, baseline body weights showed no statistical differences between wildtype and knockout mice (*Stap1*^*+/+*^ males 25.2 g +/− 3.2 vs *Stap1*^*−/−*^ males 23.5 +/− 3.1, *Stap1*^*+/+*^ females 22.5 +/− 2.6 vs *Stap1*^*−/−*^ females 19.7 +/− 1.3, *n* = 6 for each group). Body weights were measured weekly (Fig. 3a), while body composition (Fig. 3b) and tissue weights (Fig. 3c and d) were assessed the end of the WD. No significant differences were noted between *Stap1*^*−/−*^ mice over *Stap1*^*+/+*^ littermate controls in any of these parameters. Similar trends were also observed in glucose tolerance testing, monitored to ensure there were no differential changes in intermediary metabolism induced by the western diet and loss of Stap1 (Fig. 3e).

**Fig. 4:**
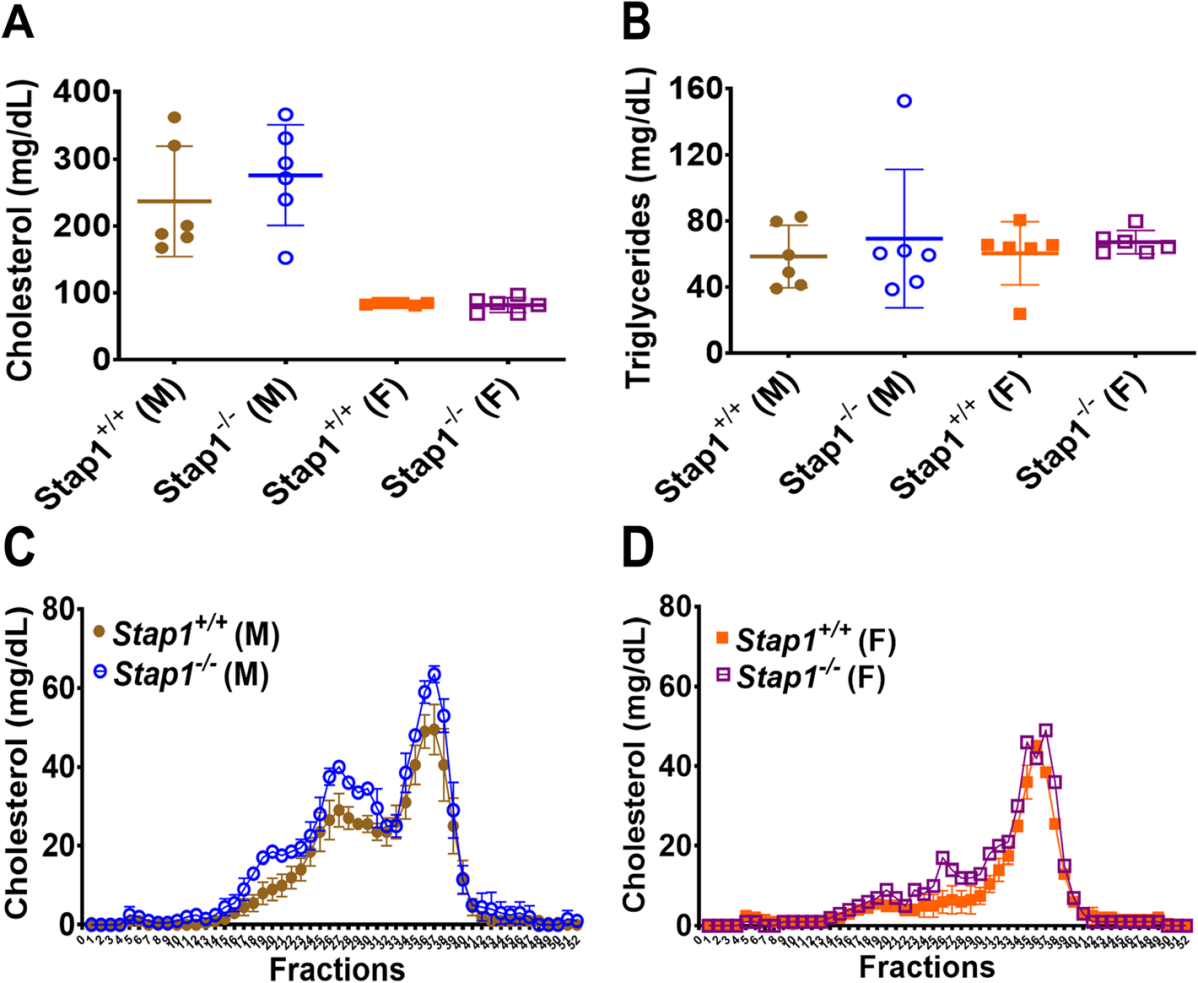
Effect of 16 weeks of western diet (WD) on plasma lipid profiles. Panel **a** shows the plasma cholesterol and panel **b** the triglyceride levels in wildtype and knockout mice (n = 6 per group). Female mice (whether wild-type or knockout) seem to be resistant to a WD-induced hypercholesterolemia (panel **a**), but this may be a feature of the mouse strain background. FPLC cholesterol profiles of the plasma (panel **c**, males, panel **d**, females) showed that the diet in males led to a dyslipidemic profile, but loss of *Stap1* did not affect these patterns. Error bars denote ±1 SD

Plasma cholesterol and triglyceride levels (Fig. 4a and b), and plasma FPLC cholesterol profiles (Fig. 4c and d) showed the diets led to a significant change in the lipoprotein distribution from baseline (compare to Fig. 2e) as expected for a high fat diet, but there were no differences between wild-type and Stap1 knockout mice.

**Fig. 5:**
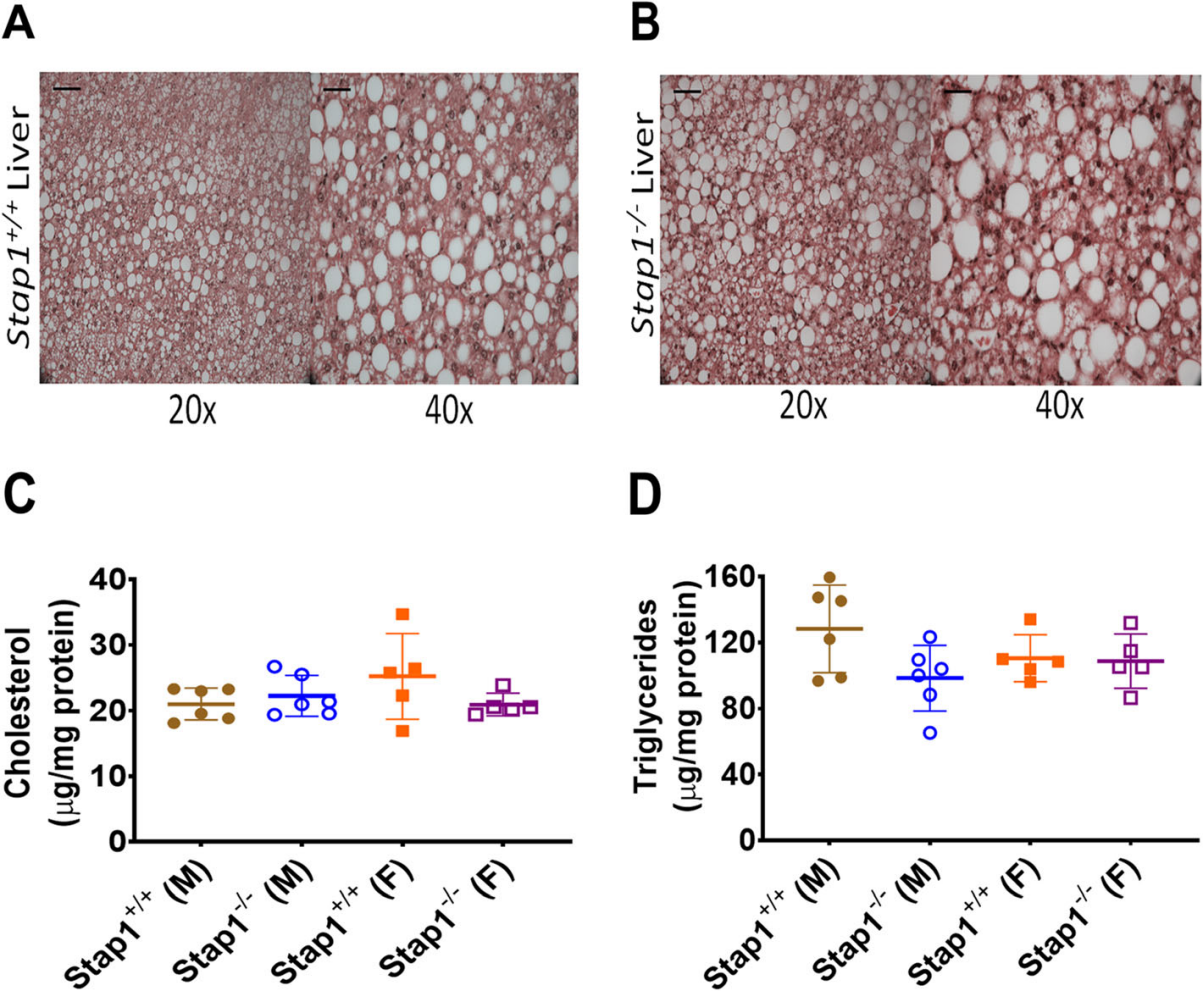
Effect of western diet (WD) on liver morphology and lipids. Assessment of liver histology by hematoxylin and eosin staining showed a fatty liver in all mice, irrespective of *Stap1* status or sex (panel **a** and **b**). This was confirmed by analyses of liver cholesterol (panel **c**, n = 6 per group in males and 5 per group in females) and triglyceride (panel **d**, n = 6 per group in males and 5 per group in females) but loss of *Stap1* does not affect these parameters. Error bars denote ±1 SD

Investigation of liver histology with hematoxylin and eosin staining (H&E) showed changes compatible with fat accumulation as would be expected after 16 weeks of WD, but found no differences in morphology between groups (Fig. 5a and b). Moreover, the elevated levels of liver total cholesterol and triglycerides (Fig. 5c and d) were comparable between WD fed *Stap1*^*+/+*^ and *Stap1*^*−/−*^ mice.

## Discussion

This study tested the hypothesis that loss of Stap1 can lead to hypercholesterolemia using *Stap1*^*−/−*^ mice. The Stap1 mice, targeted by a knockout-first, conditional targeting vector were obtained from the Welcome Trust Sanger Center and characterized. Although there is no reliable antibody for specific detection of endogenous STAP1 protein by western blot (we tested commercial antibodies from ThermoFisher and Abcam but found these unsuitable, see Supplementary files), a knockout was confirmed at the DNA and mRNA level instead. Normal Stap1 RNA expression observed in different tissues of *Stap1*^*+/+*^ mice and compared with the protein atlas database (https://www.proteinatlas.org/ENSG00000035720-STAP1/tissue); relatively high levels of expression in spleen were noted but very minimal expression was seen in liver. The higher levels of RNA also coincide with the proportional amount of *STAP1* protein in spleen of *Stap1*^*+/+*^ mice as measured through targeted proteomics [18]. QPCR analysis of livers, spleens, and kidneys of *Stap1*^*−/−*^ mice did not detect any *Stap1* mRNA, confirming the knockout (Fig. 1c). RT-PCR analysis of the mRNA produced expected amplicons when using primers targeted to exon 2 through 4 in *Stap1*^*−/−*^ mice (data not shown), but not with primers specific for exons 5, 6 or 7 (Fig. 1d) suggestive that the transcriptional message was preserved until exon 4 and was interrupted due to reporter insertion in the intronic region between exon 4 and 5. RT-PCR using primers targeted to exon 2 through 7 unexpectedly produced a product in *Stap1*^*−/−*^ mice (Fig. 1d), but sequencing showed that it was an off target product from chromosome 2 (supplemental data), whereas *Stap1* is located on chromosome 4.

Obesity and insulin resistance are commonly associated with hyperlipidemia and high caloric fat- enriched diets are widely accepted to induce hyperlipidemia [26]. A western-type diet (TD.88137) was chosen for these studies for its ability to induce hypercholesterolemia, obesity and atherosclerosis depending on the genetic background of the mice used for study [24, 27, 28]. Previous studies have shown that 16 weeks of WD regime is sufficient to induce severe hypercholesterolemia in mice [29, 30]. Augmentation of whole-body fat mass and markedly elevated circulatory cholesterol (especially males) was observed in *Stap1*^*+/+*^ and *Stap1*^*−/−*^ mice at the end of 16 weeks WD (Figs. 3 and 4). However, these changes were not exacerbated, as hypothesized, in mice lacking global *Stap1* expression and suggest the loss of Stap1 does not exacerbate hyperlipidemia.

The link between STAP1 and hypercholesterolemia was proposed when some FH4 patients were reported to have variants of the gene, in the absence of changes in known hyperlipidemia loci [6]. A review of genetic variants associated with FH noted 2580 in *LDLR*, 896 in *APOB*, 351 in *PCSK9*, 16 in *APOE*, and 4 in *STAP1* [8]. Fouchier et al in 2014 demonstrated four variants in the *STAP1* gene of FH4 patients and characterized the phenotype. All four *STAP1* variants were missense mutations (Glu97Asp, Leu69Ser, Ile71Thr, and Asp207Asn). Together with the fact that the phenotype of *STAP1* variant carriers was milder than other forms of ADH [6], one possibility is whether these are ‘false positive’ hits. Nevertheless the association between STAP1 and ADH was supported further by other studies showing elevated circulatory cholesterol in patients with *STAP1* variants [10–12, 31]. However, this link has been highly debated with studies reporting no association between *STAP1* and ADH [13–17], or no difference in plasma lipid profiles in carriers of *STAP1* variants and controls [18]. Moreover, Loaiza et al recently also found the absence of any significant difference in lipid phenotype in *Stap1*^*−/−*^ mice compared to controls, even after metabolic challenges [18]. The current study is a verification of these findings. Nevertheless, a KO mouse model is a loss of function model and does not address the possibility of a gain of function variants in *Stap1*. The fact that the previously identified *STAP1* variants were missense mutations suggests that a viable protein may potentially be expressed in those patients, though its functionality would be purely speculative.

It was initially hypothesized that extra-hepatic mechanisms were responsible for STAP1 mediated hypercholesterolemia due to the fact that liver distribution was low [6, 31]. Interestingly, recent experiments using single cell RNA-Seq indicated that its expression is highly specific to lymphoid cells (https://tabula-muris.ds.czbiohub.org/) and any expression detected by examining tissue RNA likely comes from the presence of these cells in these tissues. *Stap1* expression measured by qPCR in the current study was low in liver and other tissues except for the spleen (Fig. 1b). This could be explained by the concentration of B-cells in the spleen and their presence in other organs, as the tissues were not perfused prior to collection. Some studies have associated *STAP1* variations with cardiovascular disease [10–12], and immune cells have been linked with atherosclerosis and FH [32]. This study did not thoroughly investigate possible effects of *STAP1* deletion on the immune system, but there were no obvious changes in B-cell subsets and IgG (data not shown). Further studies would be obviously necessary to fully characterize the effects, if any, on immune function. Nevertheless, transplant of *Stap1*^*−/−*^ bone marrow (BM) into *Ldlr*^*−/−*^ female mice had no impact on plasma lipids as well on the development of atherosclerosis plaques [18]. Furthermore, there were no marked changes in B-lymphocyte populations in blood of carriers of *STAP1* gene variants compared to age- and sex-matched family controls [18].

The study by Loaiza et al was well-designed and thorough, but differs from the current investigation in two important experimental variables: 1) the strategy used to generate *Stap1* KO mice, using clustered regularly interspaced short palindromic repeats/clustered regularly interspaced short palindromic repeat-associated 9 (CRISPR/Cas9) technology vs targeted homologous recombination of embryonic stem cells and 2) the much shorter time period for hyperlipidemia induction in global and tissue-specific KO mice (4 weeks vs 12 weeks). Since 16 weeks of WD regime is sufficient to induce severe hypercholesterolemia and the fact that FH patients show elevated levels of plasma cholesterol, the long-term regime that we used is ideally advantageous over the short duration regime used in the study by Loaiza et al.

An important limitation of the study is loss-of-function models do not recapitulate the missense variants reported for STAP1 in humans. However, we are confident our current studies are valid as our characterization (in keeping with published data) finds no meaningful expression of STAP1 in cells other than B-cells. Thus, even for missense variants of *STAP1* to have a pathological role in causing hyperlipidemia in humans, a mechanistic pathway to do so would need to be identified. Based upon our current understanding of lipid metabolism, no such meaningful pathway exists.

## Conclusion

Global loss of *Stap1* in mice does not result in an abnormal lipid phenotype, and this study validates the study by Loaiza et al, who reported similar findings. Additionally, expression STAP1 mRNA seems to be confined to B-cells, suggesting alteration of lipid metabolism in a direct manner to be not feasible. Genetic changes in *STAP1* are therefore unlikely to be cause of familial hypercholesterolemia and variants reported in such rare families may represent a spurious association.

## Supplementary Information

**Additional file 1: Table S1.** Primer sequences.

**Additional file 2.** Sequencing of off-target products on chromosome 2.

**Additional file 3.**

## Data Availability

The datasets used and analyzed during the current study are available from the corresponding author upon request. Requests for mice will require a simple materials transfer agreement with the University of Cincinnati and all costs for transfer will be the responsibility of the requestor.

## Abbreviations

ADH: Autosomal dominant familial hypercholesterolemia
APOE: Apolipoprotein E
BRDG: B-cell antigen receptor downstream signaling 1 protein
FLPC: Fast Performance Liquid Chromatography
LDLR: Low density lipoprotein receptor
OGTT: Oral glucose tolerance test
PCR: Polymerase chain reaction
PCSK9: Proprotein convertase subtilisin/kexin type 9
STAP1: Signal transducing adaptor family member protein 1
WD: Western diet
