# Supplementary material for: Mice lacking global Stap1 expression do not manifest hypercholesterolemia
Source: BMC Med Genet. 2020 Nov 23;21:234. doi: 10.1186/s12881-020-01176-x (PMC7685646; doi:10.1186/s12881-020-01176-x)
Supplement: Supplementary file 1 — Additional file 1: Table S1. Primer sequences. [file 12881_2020_1176_MOESM1_ESM.docx]

**Table S1.** Primers selected for validating *Stap1* gene deletion

| Primer | Forward | Reverse | Product size (bp) |
| --- | --- | --- | --- |
| For tail snip genotyping | | | |
| *Stap1^+/+^* | GAGAACAACGAAACTAACATGAAGAGC | CTGTAGACCAGGTATTTGCCACC | 266 |
| *Stap1^-/-^* | GAGAACAACGAAACTAACATGAAGAGC | TCGTGGTATCGTTATGCGCC | 236 |
| LacZ cassette | ATCACGACGCGCTGTATC | ACATCGGGCAAATAATATCG | 108 |
| For exon splicing | | | |
| Exon 2-5 | GGACCACGCTGTTCTTTTAC  (Exon 2) | TTGCCCAGGTAGAAGTGAC  (Exon 5) | 251 |
| Exon 2-6 | GGACCACGCTGTTCTTTTAC  (Exon 2) | TTTGCTGTCACTACCAGGC  (Exon 6) | 482 |
| Exon 2-7 | GGACCACGCTGTTCTTTTAC  (Exon 2) | GAAGTGCTTGATTCTTGGC  (Exon 7) | 536 |
| For qPCR | | | |
| *Stap1* | GGAGGGGCTTCATTCTTACA | TGCCCAGGTAGAAGTGACA | 67 |
