# Supplementary material for: Mice lacking global Stap1 expression do not manifest hypercholesterolemia
Source: BMC Med Genet. 2020 Nov 23;21:234. doi: 10.1186/s12881-020-01176-x (PMC7685646; doi:10.1186/s12881-020-01176-x)
Supplement: Supplementary file 2 — Additional file 2. Sequencing of off-target products on chromosome 2. [file 12881_2020_1176_MOESM2_ESM.docx]

**Exon2 Forward sequence:**

NNNNNNATGCTGANNTGCCTTGTGTCTGATTGCATCAATGTTAACACCTTTGTTGTGCTAAAACTTTGTTAAACACCATTACTGGGGAAATTTGGGTAAAGAATACACTAAAACTCTCTCTGTGGTTTATTACAAGCAAGTATGTCTCCACATTTTTCTCCAGGTAAAATGATTTTTTTACAGTGTCAACACTGGCTTTTTGCTCACAATTTTGCATCTAGTGTTAGAAATGCACTGGCTCAGCACTACCACCAGCTGTCAGGGAAGCAGCCCCTCTGTGAGCTCACACAGCACATGAAGCAGATATTCTGCCATCTGTGCAAGGCCCTTCTCCAGGCCAGTCTTCCCTCAGACCCAGCAGGTTCTCACTAACTAATTTATTTCGATCTTTTTTTCTCACCCTCATAACATAAGCCATGGATATGTGGATATAAATGATACAAATGCTAAGTCAGCACAATTTCCTTATTCCACTCAAGTCTTTACATCACAATTATGACACTGCCAAGAATCAAGCACTTCA


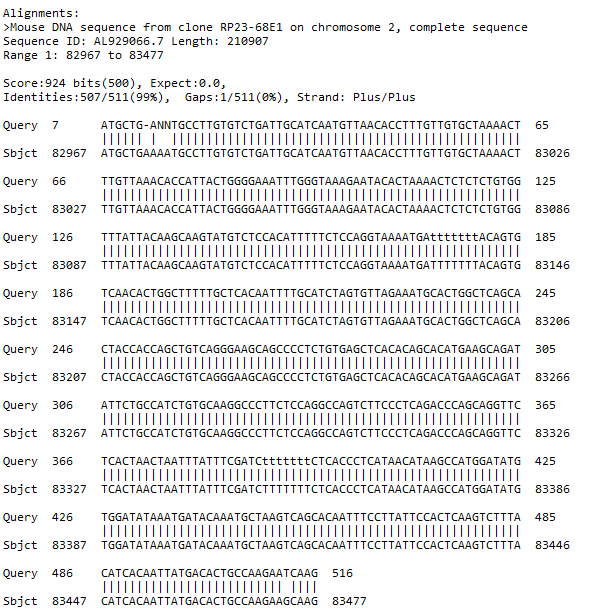
**Alignment:**

**Exon7 Reverse sequence:**

NNNNNGNNNTGNNNNNNNTTGAGTGGAATAAGGAAATTGTGCTGACTTAGCATTTGTATCATTTATATCCACATATCCATGGCTTATGTTATGAGGGTGAGAAAAAAAGATCGAAATAAATTANTTAGTGAGAACCTGCTGGGTCTGAGGGAAGACTGGCCTGGAGAAGGGCCTTGCACAGATGGCAGAATATCTGCTTCATGTGCTGTGTGAGCTCACAGAGGGGCTGCTTCCCTGACAGCTGGTGGTAGTGCTGAGCCAGTGCATTTCTAACACTAGATGCAAAATTGTGAGCAAAAAGCCAGTGTTGACACTGTAAAAAAATCATTTTACCTGGAGAAAAATGTGGAGACATACTTGCTTGTAATAAACCACAGAGAGAGTTTTAGTGTATTCTTTACCCAAATTTCCCCAGTAATGGTGTTTAACAAAGTTTTAGCACAACAAAGGTGTTAACATTGATGCAATCAGACACAAGGCATTTTCAGCATCAAAGACTCCCTCCTCTGGTAAAAGAACAGCGTGGTCCANN


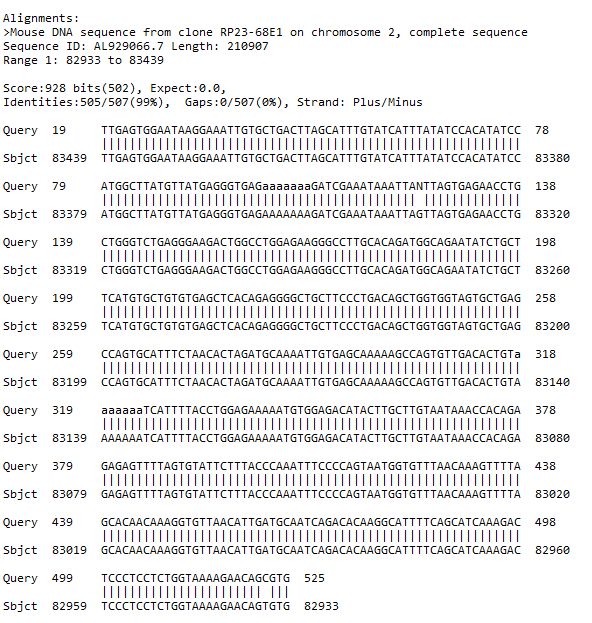
**Alignment:**
