# Supplementary material for: Mice lacking global Stap1 expression do not manifest hypercholesterolemia
Source: BMC Med Genet. 2020 Nov 23;21:234. doi: 10.1186/s12881-020-01176-x (PMC7685646; doi:10.1186/s12881-020-01176-x)
Supplement: Supplementary file 3 — Additional file 3. [file 12881_2020_1176_MOESM3_ESM.docx]

Western Blot analyses using two commercially available anti-STAP1 antibodies


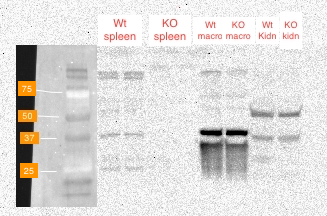

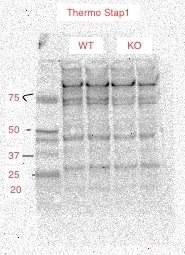


The left panel shows the result using antibody obtained from ABCAM (ab103646, Cambridge, UK) and the right panel shows the result from ThermoFisher Scientific (PA5-11545, USA). The molecular weight markers are as indicated and the predicted molecular weight of mouse Stap1 is 34.6kDa. Expression of Stap1 is expected in spleen but in kidney. The Thermo antibody was used in samples from spleen only and while a band is expected in samples from wildetype (WT) spleen, none was observed. Although a band was seen with the knockout tissues (KO) with the ABCAM, a band was seen in tissues not expected to express Stap 1 (kidney) or macrophages (macro).
